# Recruitment of young adults into a randomized controlled trial of weight gain prevention: message development, methods, and cost

**DOI:** 10.1186/1745-6215-15-326

**Published:** 2014-08-16

**Authors:** Deborah F Tate, Jessica G LaRose, Leah P Griffin, Karen E Erickson, Erica F Robichaud, Letitia Perdue, Mark A Espeland, Rena R Wing

**Affiliations:** The University of North Carolina at Chapel Hill, Chapel Hill, NC USA; School of Medicine, Virginia Commonwealth University, One Capitol Square, 9th floor, Richmond, VA 23298 USA; Wake Forest University School of Medicine, Medical Center Boulevard, Winston-Salem, NC 27157 USA; The Miriam Hospital/Weight Control and Diabetes Research Center, 196 Richmond Street, Providence, RI 02903 USA; Department of Psychiatry and Human Behavior, Warren Alpert Medical School of Brown University, Providence, RI USA; Gillings School of Global Public Health, UNC-Chapel Hill, Rosenau Hall, Campus Box 7440, Chapel Hill, NC 27599-7440 USA

**Keywords:** weight gain prevention, young adults, recruitment methods, cost

## Abstract

**Background:**

Young adulthood (age 18 to 35) is a high-risk period for unhealthy weight gain. Few studies have recruited for prevention of weight gain, particularly in young adults. This paper describes the recruitment protocol used in the Study of Novel Approaches to Prevention (SNAP).

**Methods:**

We conducted extensive formative work to inform recruitment methods and message development. We worked with a professional marketing firm to synthesize major themes and subsequently develop age-appropriate messages for recruitment. A variety of approaches and channels were used across two clinical centers to recruit young adults who were normal or overweight (body mass index (BMI) 21 to 30 kg/m^2^) for a 3-year intervention designed to prevent weight gain. We tracked recruitment methods, yields, and costs by method. Logistic regression was used to identify recruitment methods that had the highest relative yield for subgroups of interest with covariate adjustments for clinic.

**Results:**

The final sample of 599 participants (27% minority, 22% male) was recruited over a 19-month period of sustained efforts. About 10% of those who initially expressed interest via a screening website were randomized. The most common reason for ineligibility was already being obese (BMI >30 kg/m^2^). The top two methods for recruitment were mass mailing followed by email; together they were cited by 62% of those recruited. Television, radio, paid print advertising, flyers and community events each yielded fewer than 10% of study participants. Email was the most cost-effective method per study participant recruited.

**Conclusions:**

These findings can guide future efforts to recruit young adults and for trials targeting weight gain prevention.

**Trial registration:**

ClinicalTrials.gov NCT01183689 (registered 13 August 2010).

## Background

Young adulthood is a high-risk period for unhealthy weight-related behaviors, weight gain, and obesity [1, 2]. On average, young adults gain 1 to 2 pounds per year [3], which is associated with increased cardiovascular risk and metabolic syndrome [4]. Approximately half of adults <35 years old are already overweight or obese, placing them at risk for additional weight gain [2]. Thus, effective weight gain prevention may be critical to reducing disease risk and improving overall health.

Despite a clear need, significant challenges exist related to recruitment of young adults both for research studies and for clinical programs related to weight control. First, young adults are less likely to participate in behavioral weight control trials and those who enroll are less likely to be retained [5]. Second, prevention may be a difficult ‘sell’, given that primary prevention programs are asking individuals to change behavior when they may not perceive there is a problem to address. In the case of young adults, this is expected to be challenging given that health consequences of gaining weight and obesity are likely more distant [6].

While the challenges of recruiting young adults have been documented [5, 6], there are limited data to help inform recruitment efforts for weight gain prevention studies. Previous studies suggest that recruitment for weight gain prevention may prove more challenging than recruiting for weight loss. A study of women 25 to 44 years old with a body mass index (BMI) of 21 to 30 kg/m^2^, found that almost half of the women who expressed interest were ineligible largely due to BMI being too high, and another 30 to 40% of those screened were not interested [7]. Similarly, recruitment of families with young children for weight gain prevention has proven challenging and relatively costly, with estimates of over $350 required to recruit each family [8]. These data suggest that recruitment for weight gain prevention studies may require significant attention and resources to identify and enroll interested and eligible participants.

In the current paper, we describe the development of the recruitment messages and approach, as well as the costs and yields of the recruitment methods, used in the Study of Novel Approaches to Prevention (SNAP) trial. SNAP is a randomized clinical trial designed to test two different approaches to weight gain prevention compared to a control in adults aged 18 to 35 over an average planned follow-up of 3 years. The protocol for the study has been published previously [9]. Briefly, the two interventions being tested are both based on a self-regulation model that has been shown to help prevent weight regain in recent weight losers [10]; however, the underlying targets and approach of the interventions differ substantially. One self-regulation intervention is focused on making small, consistent, daily changes in eating and exercise behavior to prevent weight gain; the other emphasizes periodic, short term, larger changes in eating and exercise behavior that result in small weight losses to buffer future gains.

The study planned to recruit and enroll 600 adults (300 at each the clinical site), aged 18 to 35 years old and BMI 21 to 30 kg/m^2^, with goals of 25% men and 25% racial or ethnic minorities - goals established jointly by the research team and funding agency. Participants were recruited from August 2010 to February 2012 and will be followed for 24 to 48 months (mean = 3 years). The goals of this paper are: 1) to describe the development of recruitment messages and a general recruitment plan specific to young adults for weight gain prevention, 2) to present data on recruitment strategies used and their yield, and 3) to report the costs associated with recruitment strategies used. To our knowledge, this is the first study documenting not only the formative data informing message development but also costs and yield of specific recruitment strategies within a weight gain prevention trial for young adults, which may be useful for other studies in this population, on this topic, and for clinical trials in general.

## Methods

The project, funded by the National Heart Lung and Blood Institute, involves two clinical centers (Miriam Hospital/Alpert Medical School of Brown University and the University of North Carolina at Chapel Hill) and a Data Coordinating Center at Wake Forest University School of Medicine. All procedures were reviewed and approved by the Institutional Review Board (IRB) at each site (Rhode Island: The Miriam Hospital (Lifespan) IRB #2018-07; University of North Carolina at Chapel Hill IRB #07-1783; Wake Forest: Wake Forest School of Medicine IRB #00010097) and study participants gave written informed consent at each clinical center.

### Formative research and message development

Based on the experiences with recruiting a similar population for a pilot study [11] and previous research on weight gain prevention [7, 8], we anticipated challenges with recruitment for a weight gain prevention program targeting young adults. Thus, we designed a formative research phase to inform our recruitment plan and intervention efforts for SNAP. Qualitative research methods, such as focus groups, offer a unique opportunity to formally assess the perceptions, needs and preferences of young adults who would be eligible for a weight gain prevention trial, but who may not otherwise present for treatment. Although a detailed description of methods, analyses and findings is outside the scope of the present paper, we present a brief overview of methods below, as well as key themes that informed our recruitment efforts in the present trial.

Focus groups were conducted at both clinical sites (Providence, RI and Chapel Hill, NC) in November of 2009. Participants were recruited using a multimethod, community-based approach including email blasts and posts to listservs, ads in college newspapers and e-newsletters, on-air radio spots, local newspapers, and internal hospital and university intranets. Interested participants completed a brief phone screen with study staff to ensure eligibility (18 to 35 years of age with a BMI of 21 to 30 kg/m^2^). Those who remained interested and were eligible were invited to participate in the next available focus group. The primary aim of the focus groups was to help researchers understand how to make weight gain prevention programs more appealing to this age group. Groups were conducted by staff members with experience leading behavioral weight control interventions and/or trained in qualitative data collection and lasted approximately 90 minutes; a facilitator’s guide was developed to ensure consistency across groups and sites. The agenda focused on three key areas: 1) perceptions of weight gain/determining whether 18 to 35 year olds viewed the potential for weight gain as a problem; 2) recruitment messages (that is, how to frame prevention/‘sell’ it to this age group); 3) recruitment outlets (that is, where should we advertise and what mediums should we use). All participants provided informed consent and were paid $20 for participation.

Across sites we conducted a total of 14 groups (nine all-female groups, four all-male, and one mixed sex) before achieving theoretical saturation. Participants (n = 68; 32% male; 68% female) were mostly non-Hispanic whites (58.4%), with a mean age of 26.1 ± 4.85 years and a mean BMI of 24.69 ± 2.71 kg/m^2^. Groups were audio-taped and executive summaries were prepared that summarized major themes within each agenda section, accompanied by interpretations by researchers as to the implications for the study. The executive summaries were compiled using a combination of debriefing summaries and structured audio reviews from each group.

Completion of the focus groups influenced our subsequent approach to recruitment by highlighting several themes. First, there was recognition of weight gain as a problem as one ages. Groups noted that highlighting the statistics among young adults would be of benefit to raise awareness. They also indicated that the cumulative effect of weight gain over time was perceived as more powerful and motivating than hearing about the expected year-to-year gains. For example, using the tag line or message ‘young adults gain an average of 20 pounds over 10 years’ was perceived as more influential than ‘young adults gain 1 to 2 pounds per year’. The second theme that emerged was consensus across groups that to capture young adults’ attention, messages should be positive and brief, with an emphasis on ability to produce positive changes and take action. This is consistent with efficacy promoting messages. Groups noted that they would prefer to visit a recruitment website than to call for information and indicated that a website address might be more easily remembered than a phone number and should be highlighted. Groups preferred using a specific age range in ads as opposed to ‘young adults’, using bright colors and realistic images, and avoiding an exclusive focus on images of scales. Finally, all groups generally noted that online recruitment methods would be effective, particularly email-based recruitment. They reported that ads should be primarily placed in online versions of papers as opposed to print and that a social media presence for the study might be helpful. Notably, they reported being unlikely to click on paid advertising placed on social networking sites (for example, Facebook).

### Recruitment plan

**Figure 1:**
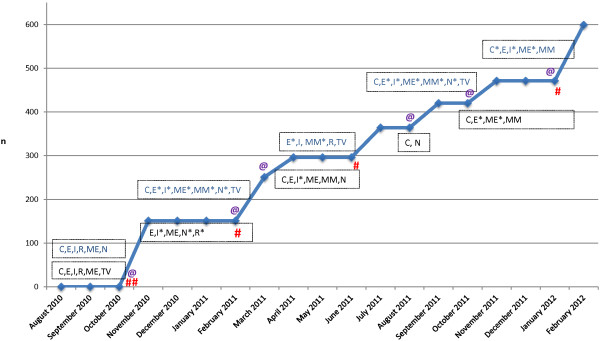
**Major recruitment events May 2010 to March 2012.** C, community events; E, emails or mailed letters to individuals or smaller groups; I, internet ad or website posting; ME, mass email to listserv or purchased email list; MM, mass mailing; N, newspaper ad; R, radio ad; TV, television ad. (* denotes multiple runs during period). Not shown: flyers posted or “word of mouth”. Events shown in blue were in Providence and those in black were done in North Carolina. Symbols indicate when a cohort was randomized at each location: # in North Carolina; @ in Providence.

Following the formative phase, a recruitment plan was devised. The strategy began by hiring a professional marketing firm that provided recommendations for branding, marketing and advertising based on the findings of our formative work. We selected recruitment channels that had been successfully used in other studies, were suggested by the focus groups, and reached our target demographic based on data available from the media outlets (for example, TV stations chosen based on their viewership). Finally, our approach was chosen to balance cost with opportunities to recruit a more diverse group. For example, while free email listservs were convenient and appealing, direct mail was deemed likely to enable us to reach a more economically diverse potential participant pool, so both methods were used. Special attention was paid to developing community relationships within organizations that might enable recruitment of men and minorities. Recruitment was also planned in cohorts but generally did not stop between cohorts. The timing and use of specific channels of recruitment can be seen in Figure 1. Our recruitment plan included careful real-time tracking of recruitment methods, which enabled us to prioritize channels with successful yields to recruit subsequent cohorts.

### Recruitment message development

**Figure 2:**
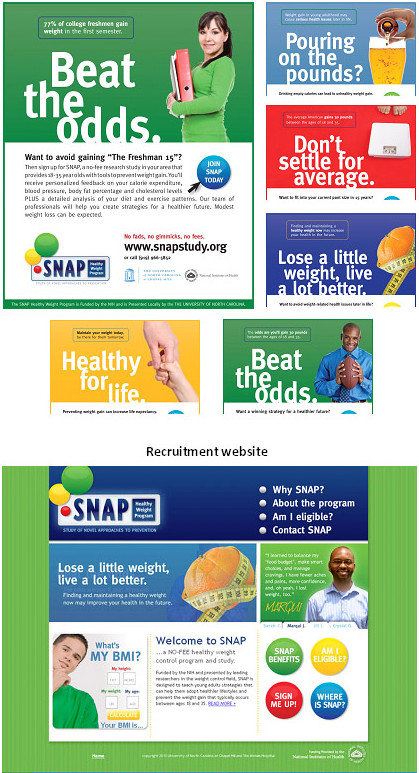
**Recruitment materials.**

Findings from the focus groups were provided to a professional marketing firm and were used to develop recruitment messages, create a project brand, produce a variety of recruitment materials, and design a recruitment website (see Figure 2). Branding of the study included a study logo, colors, fonts and graphic elements that were consistently used across channels and sites. Specific attention was paid to attracting a diverse group of young adults. General taglines to appeal to a broad spectrum of our target demographic were created with segmented advertising taglines to attract specific market segments or sub-groups of young adults within the eligible demographic. Recruitment of males and minority individuals was a priority as both have been underrepresented in prior studies of weight control [1, 12–14], and our funding guidelines encouraged minimum levels of diversity in the study sample of at least 25%. To encourage male and African American recruitment, images of individuals from these demographic groups were used. Figure 2 provides example messages.

All recruitment advertising referred participants to the website for more information about the study and to complete initial eligibility screening (prescreen). The recruitment website included description of the study, benefits of participating, testimonials with pictures from four participants in the pilot study [11], eligibility criteria (including a BMI calculator), study locations, and the link to complete the online prescreening questionnaire. Recruitment was conducted simultaneously in RI and in NC using the same general messaging, formats and materials but with flexibility to focus more on specific outlets or techniques that seemed to be producing the greatest yield.

Recruitment was conducted from August 2010 through February 2012 (see Figure 1) through a broad variety of mechanisms including mass mailings, email listservs, newspaper, radio, television, websites, word of mouth, *etcetera*. During telephone screening, participants were asked to report how they heard about the study, and method of recruitment was tracked. A small number of participants (n = 15) reported more than one recruitment method. For purposes of estimating costs for recruitment methods in this paper, a primary method was determined for each person, defaulting to those with greater cost. The procedures for each method are described in more detail below.

### Recruitment strategies

#### Mass mailing

Mailing lists were obtained from USA Data for men and women ages 18 to 35 within a 30 to 50 mile radius of Chapel Hill, NC and Providence, RI. Over a one-year period, a total of 30,000 postcards and 318,176 brochures were mailed in batches of 30,000 to 50,000. Half of the postcards specifically added ‘Men:’ to the tagline to target males. The postcard included a very brief description of the study and directed those interested to the study website. A variety of brochure designs was created to appeal to sub-populations within the targeted demographic.

#### Email

Emails were sent to email listservs from affiliated organizations, contact individuals within various organizations that employed or had members in the target demographic, and purchased email lists. Html emails were sent as well as text-based versions. The majority of emails were sent at no cost. Listservs included university and hospital student and staff lists and varied across the two clinical locations based on the availability of listservs and the presence of organizations that could yield interested participants. Other email recipients (most requesting that the announcement be forwarded to constituents) included human resources directors, wellness coordinators, local business contacts, employee resource groups, and university leaders. Groups that may have a significant contingent of young men (for example, police and fire departments, builders, and barbers) and African Americans (for example, historically black colleges and African American alumni and student groups) were specifically included. Emails were also sent to former study participants and current SNAP study participants. An additional email strategy was tried during recruitment of the last cohorts at each site where email lists were purchased from USA Data. Emails from the purchased lists were sent to 24,714 email addresses. The decision to purchase email lists was made during the last cohort of recruitment since our free email list resources had been largely saturated. The list was purchased from the company from whom we had purchased the print mailing lists since that strategy had met with some success.

#### Print advertising

Print news ads ran in city and town papers and magazines in the Providence and Chapel Hill areas, as well as campus newspapers, newsletters, and magazines. A total of four ads were placed in NC with runs of 1 day or 1 issue to 1 week. In RI, 35 ads were placed with similar frequency. The study was also featured for free in newspaper articles related to health and obesity at each location.

#### Television

Television ads were placed on local cable stations in the Providence, RI and Chapel Hill/Raleigh/Durham, NC viewing areas. In NC, a total of 280 thirty-second ads were run on a variety of stations including local news, sports, and lifestyle channels. In RI, approximately 600 ads were run on similar stations. The study was also featured on a local news channel interview in RI. Television ads in both locations ran for 2- to 6-week periods over 9 months.

#### Radio

Thirty second radio advertisements were placed on a variety of popular location stations including music, talk and sports radio. A total of 451 spots were run over 5 months.

#### Website

Website ads (both paid and unpaid) were posted on websites including online newspapers, study clinic websites, university clinical trials advertising, online classifieds (Craigslist), hospital and university intranet, and health-related organization websites.

#### Other

Other recruitment methods included posting flyers and posters in the surrounding areas in public places and in relevant locations such as fitness clubs, libraries, auto parts stores, medical offices and university campuses. Information tables were set up at community events such as health fairs, on university campuses, at farmer’s markets, and at races. At the beginning of the study, a press release was issued in both study areas. Another study with young adults was recruiting simultaneously in the Durham metro area of North Carolina at another institution. The other study was for weight loss rather than weight gain prevention; therefore, the eligibility criteria for BMI for the two studies were not identical. Investigators from the two studies met and devised a strategy to make referrals from each study to the other for ineligible participants. As recruitment spread, word of mouth was reported as an additional recruitment method.

### Statistical analysis

Descriptive statistics for continuous measures are provided with means and standard deviations. Counts and percentages are provided for the categorical variables. Logistic regression was used to identify recruitment methods that had the highest relative yield for demographic and BMI subgroups, with covariate adjustment for clinic.

## Results

**Figure 3:**
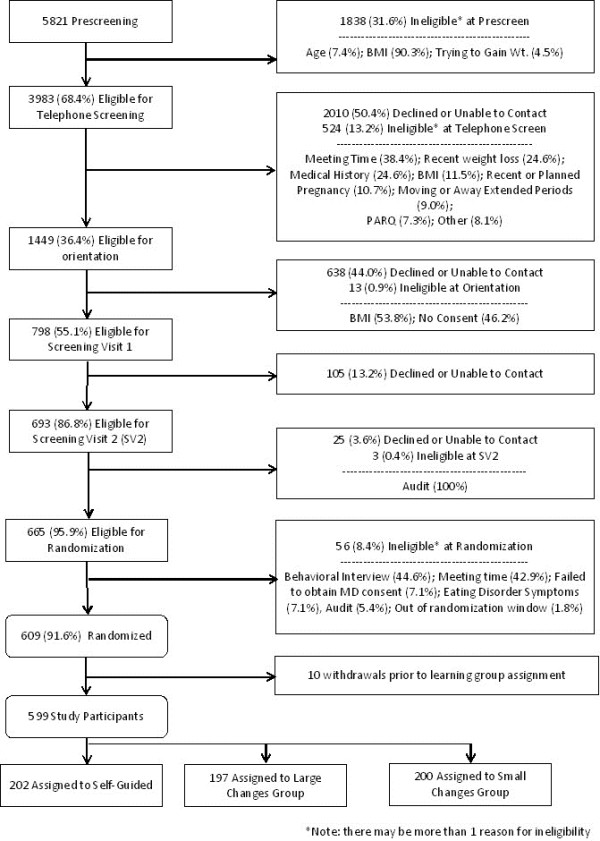
**CONSORT diagram.**

The CONSORT diagram (Figure 3) indicates the number screened and the reasons for ineligibility at each point in the recruitment screening process. Online prescreening forms (n = 5,821) were completed on the study recruitment website. Age, BMI and intentions for purposeful weight gain were recorded, and almost 70% of those completing the online screener were eligible based on these minimal criteria. Of those ineligible at prescreen, the majority (90%) were due to BMI, and of those excluded for BMI, 95% were for BMI above 30 kg/m^2^.

Of those eligible on the website prescreen (n = 3,983), approximately 50% (n = 1,973) were subsequently reached for telephone screenings to further determine eligibility. Although multiple reasons for ineligibility could be endorsed, the three most common reasons for ineligibility during the telephone screening were 1) unable to attend the meeting time (38.4%), 2) weight loss of more than 10 lbs in the past 6 months (24.6%), and 3) medical reasons (24.6%).

About half (56%) of those who remained eligible after the phone screen attended an orientation. At orientation, 13 people were ineligible due to measured BMI being out of range (n = 7) or because they declined consent (n = 6). Subsequently, another 130 individuals did not schedule their measurement visits; this was the major reason for lack of randomization following orientation.

A total of 609 met criteria for randomization and were randomized; thus 10.5% of the 5,821 who were initially screened were randomized. An additional ten people never attended the randomization visit and did not learn of their group assignment. The study sample included 599 of the targeted 600 participants randomized across the three study treatment groups. Demographic characteristics for the final sample are presented in Table 1. On average, participants were 28.2 (4.4) years of age and had a BMI (kg/m^2^) of 25.4 (2.6); 46.2% were normal weight (BMI 18 to 24.99) and the others were overweight. Of the sample, 21.7% were males, 26.9% were minorities, and 95.7% had at least some college.Figure 1 shows the actual randomization by month over the recruitment period from August 2010 to February 2012. Of note is the fact that the interventions were offered in groups of approximately 13 to 25; thus, recruitment occurred in waves at each clinical site, with a goal of randomizing 45 to 60 participants (and allocating to one of the three intervention conditions) in each wave per site.

**Table 1:** **Baseline demographic characteristics: overall and by site**

		All randomized N = 599 N(%)	Providence N = 292 N(%)	University of North Carolina N = 307 N(%)
**Age**	**18-25**	213(35.6)	97(33.2)	116(37.8)
	**26-35**	386(64.4)	195(66.8)	191(62.2)
**Sex**	**Male**	130(21.7)	59(20.2)	71(23.1)
	**Female**	469(78.3)	233(79.8)	236(76.9)
**Race**	**African American**	66(11.0)	15(5.1)	51(16.6)
	**American Indian**	0(0)	0(0.0)	0(0.0)
	**Asian/Pacific Islander**	24(4.0)	7(2.4)	17(5.5)
	**White (Non-Hispanic)**	438(73.1)	227(77.7)	211(68.7)
	**Hispanic**	46(7.7)	28(9.6)	18(5.9)
	**Other/Mixed**	25(4.2)	15(5.1)	10(3.3)
**Education**	**<High school graduate**	1(0.2)	0(0.0)	1(0.3)
	**High school graduate**	25(4.2)	15(5.1)	10(3.3)
	**Any college**	372(62.1)	192(65.8)	180(58.6)
	**Post college**	201(33.6)	85(29.1)	116(37.8)
**Body mass index (kg/m** ^**2**^ **)**	**<25**	277(46.2)	104(35.6)	173(56.4)
	**≥25**	322(53.8)	188(64.4)	134(43.6)

Overall, 38.4% of the final sample indicated that they heard about the study through mass mailing. Email was identified by 23.2% and word of mouth by 12.7% of randomized participants. Across all demographic groups, television, radio, paid print advertising, flyers and community events each yielded fewer than 10% of study participants.

### Recruitment methods for specific subgroups

**Figure 4:**
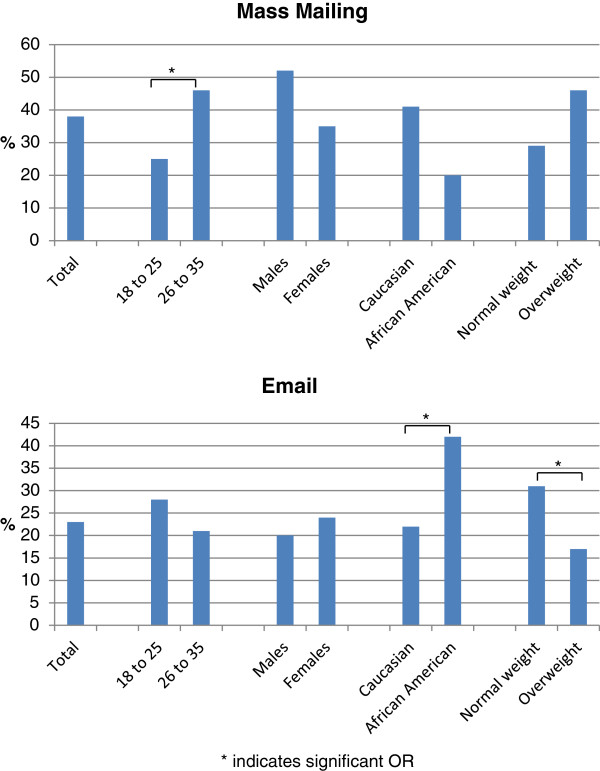
**Recruitment of subgroups by mass mailing and email.** Note: percent recruited with mass mailing and email within subgroups will not add to 100% as those recruited with other methods are not displayed.

The top two methods reported for recruitment of the final study sample are presented in Figure 4 by age, sex, race and BMI at study entry. Logistic regression models demonstrated that certain recruitment approaches were more effective in recruiting subgroups of participants. Mass mailing was identified as the recruitment source by a greater proportion of the older compared to younger participants: OR = 2.84 (1.89, 4.48). Email/listserv was noted by more African-American participants compared to non-Hispanic whites: OR = 1.99 (1.12, 3.55) and by more normal weight compared to overweight participants: OR = 1.82 (1.19, 2.78). Word of mouth was more effective in recruiting women: OR = 2.11 (1.04, 4.29), younger individuals: OR = 2.03 (1.23, 3.35) and overweight individuals: OR = 1.93 (1.13, 3.30). Flyers and newspapers each attracted very few participants, but flyers were relatively more successful for younger individuals: OR = 2.64 (1.20, 5.81) and newspapers for normal weight, compared to overweight individuals: OR = 3.92 (1.00, 15.07).

Table 2 provides the recruitment yield by cost and is ordered from the largest to least cost per participant recruited. Of note, expenses include those to pay for advertising, print brochures, create websites, *etcetera*; however, personnel costs are not included. Personnel costs are unavailable as they were not tracked by method and were unable to be separated from other study activities. Thus, in the email category, costs included those to purchase an email list, but not those incurred for a staff member to call a Human Resources manager to inquire about sending an email that could be forwarded to their listserv, nor time incurred in drafting an email. The most costly recruitment method per randomized participant was television, which was over $1,000 per randomized participant, followed by traditional print advertising, then radio. Mass mailing, which was very costly overall, had a fairly high yield, which brought the cost per recruited individual to approximately $330. Of paid methods of recruitment, use of email/listservs was the most affordable at a cost of approximately $37.50 per randomized participant, though many listservs did not have associated fees.

**Table 2:** **Recruitment methods, yields and associated costs**

	Total cost ^a^	Participants recruited with method N(%)	Cost per participant recruited
Television	$24,074.00	22(3.7%)	$1,094.27
Print Media (local newspapers, campus newspapers, magazines)	$10,555.88	13 (2.2%)	$811.99
Radio	$15,262.00	24 (4.0%)	$635.92
Mass mailing (mailing list, printing, postage)	$76,466.34	230 (38.4%)	$332.46
Website recruitment (includes both free website postings and paid web advertising)	$5,222.23	54 (9.0%)	$96.71
Email	$5,250.00	139 (23.2%)	$37.77
Flyers and community events	$2,713.27	28 (4.7%)	$96.90
Study referral	n/a	12 (2.0%)	n/a
Word of mouth	n/a	76 (12.7%)	n/a
Other: Did not report/unknown	n/a	1 (0.1%)	n/a
TOTAL	$139,543.72	599 (100%)	$232.96

^a^Personnel costs are not included.

## Discussion

The SNAP study, targeting weight gain prevention in 18 to 35 year olds, was successful in recruiting 599 participants over a 19-month period. As expected, recruitment required significant and sustained efforts; only 10% of those prescreened for eligibility and 19% of those who completed a telephone screen were retained in the final study sample. The final recruited sample was 27% minority and 22% male, with 46% normal weight and 54% overweight based on measured BMI.

Several recently published randomized weight loss trials have provided information on recruitment yields. These ranged from 9 to 12% for special populations (for example, partners, consumers of specific beverages or snack foods) [15–17] to 19% for Look Ahead [18], 20% for Premier [19], and 21% for general population volunteers [20] for weight loss. Fewer studies have been conducted on weight gain prevention [21–24]. Levine *et al*. [7] reported that of 1,816 women age 25 to 44 screened for a weight gain prevention trial, 15.6% were randomized. In a study of weight gain prevention among premenopausal women age 44 to 50, 25% of those initially screened were randomized [22]. Somewhat lower yields were shown in a study conducted among mothers of school-aged children in Australia [23]; of 2,530 women invited to participate, 10% were randomized. Finally, in a small pilot [11] we conducted in this age group, 22% of those who were telephone screened were randomized.

In the current study, the leading reason for ineligibility was already being obese. Over 90% of individuals who were ineligible during the web-based prescreen were ineligible due to a BMI >30 and over half of participants randomized into SNAP were overweight. The Australian weight gain prevention study in mothers did not exclude for BMI, and 60 to 70% were overweight or obese with 27% of the sample having a BMI >30 [23]. Obesity has been reported by others as the leading reason for ineligibility for weight gain prevention trials [7], including in one of the few weight gain prevention studies conducted in men [24]. Levine [7] also reported that 38% of those who inquired about the study declined further screening due to lack of interest. These data underscore the difficulty of ‘selling’ prevention to those of normal weight, and the fact that our recruitment messages appear to attract a high number of individuals who are already overweight or obese. The Health Belief Model [25] posits that an individual must first perceive susceptibility and believe a threat is severe enough to warrant action. It is likely that for many young adults the perceived threat of gaining weight is low, given the health consequences associated with weight gain are distant. Given that weight gains during young adulthood are estimated to be 30 lbs and sustained weight loss is challenging [26], prevention of weight gain for normal weight and preventing further weight gain for those already overweight appears important. Future research should explore how prevention messages can be adapted to be more salient to young adults and others of normal weight. It may be helpful to develop messages and test them through formal experiments to determine which messages produce the greatest recruitment yield or interest, particularly among young adults who are normal weight and may not perceive themselves as at risk.

In SNAP, specific recruitment goals were established for men and minorities: 25% men and 25% minorities. Efforts to recruit both included purchasing mailing lists in specific zip codes, purchasing advertising on radio or television stations and in print media that were popular with or targeted to those demographics, establishing relationships with community partners and attending community events geared to each special population, and working with a professional marketing company to design ads to appeal to the age and demographic segments based on findings from the formative phase of this trial. We were successful at recruiting 27% minority, but only recruited 22% men. We had particular difficulty recruiting minority males; only 17 percent of minority participants (or 4.5% of the total SNAP sample) were minority males. Pound of Prevention, one of the larger weight gain prevention studies in adults, showed similar recruitment of men with 20% male. The challenges we experienced recruiting young men are consistent with previous findings. LaRose *et al*. [27] found that young men were less concerned about weight gain compared with young women, and would have to gain 4.5 kilograms before taking action. Further, men reported being less willing than women to join a weight control program [27]. Based on our formative work and our pilot study, men describe different goals for their weight, focused less on being in a particular weight range but rather on becoming more muscular or improving fitness. Future work is needed to understand what techniques might attract men and other underrepresented groups to weight control studies.

The breakdown in ages of young adults recruited for SNAP shows that only about one-third (36%) of participants in SNAP were ≤25 years of age. Previous findings indicate that this younger range of young adults, often referred to as ‘emerging adults’ [28], is particularly challenging to recruit. In previous studies, 18- to 25-year olds were rarely recruited in standard behavioral weight loss trials and are underrepresented even relative to young adults in general (that is, ≤35 years) [5]. This is concerning given the significant weight gain observed during the early twenties [2].

Recruitment methods used in SNAP were diverse and varied by clinical site based on opportunities and also on yield. The greatest proportion of participants in SNAP was recruited via mass mailings and email/listserv. Mass mailings accounted for 38% of the recruits and although this approach was costly, the high yield made it a relatively cost-effective approach at $330 per participant. Email was the second most fruitful method for recruiting young adults with 23% reporting this method. Email was clearly most cost-effective with a cost of only $38 per recruited participant. Interestingly, the majority of the cost for email was incurred by purchasing the USA Data lists used to recruit our last cohorts. Open and click through data indicated that almost no one accessed our information (open rates) and click through to our website among those opening the emails was also very low. Thus, the majority of persons recruited via email into this trial were through free email lists and blasts that were either accessible as student or employee listservs, and others accessed via networking with wellness and nutrition professionals, local businesses, human resource directors, *etcetera*. The direct outlay cost for email recruitment may be even lower than our projections indicate. We also yielded 2% of our study sample using another free strategy by dovetailing efforts with investigators recruiting this age group for another weight-related trial in close proximity to one of the clinical sites. Investigators might consider this strategy if there is an opportunity to maximize recruitment while minimizing costs for multiple studies.

Contrary to recruitment for our previous weight control studies, newspaper, television, and radio advertising yielded fewer than 10% of recruited study participants and cost between $500 per participant recruited from radio to over $1,100 per participant recruited from television advertising. This lower yield may reflect different media use for 18- to 35-year olds compared to participants in most standard behavioral weight loss programs who tend to have average age in the mid-40s. It is also possible that while weight loss may be something that people are used to hearing about on television or radio, weight gain prevention is a concept that may be more difficult to convey or to capture the intended audience’s attention.

Overall recruitment costs for SNAP are difficult to compare to the overall literature as no large studies have been conducted in young adults or in weight gain prevention that have include detailed breakdown of costs for recruitment by method. The average cost per participant recruited and successfully randomized across all methods in SNAP was about $233. This cost is somewhat in line with other studies though direct comparisons are limited by differences in populations and methods used. For example, a study of families with young children found average costs to be about $100 higher per participant than SNAP, but their evaluation included personnel cost, so costs are likely comparable [8]. A study on minority recruitment in the Women’s Health Trial reported costs excluding personnel, that varied by region of the country and recruitment channel but generally found mass mailing costs per yield to range from $100 to $144 (in 1998) per randomized participant and to be well below those of television and other mass media. Comparison of SNAPs costs and yields with those of other trials will become more comparable as investigators routinely track and publish these data.

Exploratory analyses indicated some evidence that different approaches were more effective for recruiting different groups of participants. Specifically we found that mass mailing was more successful for recruiting older individuals, and younger participants cited flyers and word of mouth. Email was more effective in recruiting normal weight, compared to overweight participants, and African Americans compared to Caucasians. Newspapers appeared to be relatively more successful for recruiting normal weight individuals compared with overweight, but the overall yields were very low.

These findings are subject to several limitations. Recruitment outlets and messages were employed across both clinical sites, and those within each site that were most fruitful were continued. Thus, the overall yield for each outlet is influenced by the extent to which these methods were employed. Also, similar recruitment messages were deployed broadly across outlets so yield of specific messages was difficult to track. As such, no data are available as to which of the messages used in the SNAP trial may have been most salient to young adults overall, or to specific subsets of the population. The field would benefit from well-executed recruitment experiments to better elucidate which messages are most effective. Finally, recruitment messages and outlets were determined in large part by formative work conducted at the clinical sites, and data presented here reflect recruitment yield and cost at these same clinical sites; findings may not generalize to young adults in different regions of the country.

With the above limitations noted, the current study represents a unique contribution to the literature. Few large-scale trials have targeted young adults for the prevention of weight gain. Very little is known about the yield and costs of specific recruitment approaches in clinical trials. Based on recruitment challenges in our pilot study [11], we allocated ample time and resources to enable successful recruitment of a challenging population. The SNAP trial undertook extensive formative work, which guided message development and recruitment planning and was critical to the design of effective communications and program offerings that would be appealing to this population. Hiring marketing experts was beneficial to produce high quality materials and a relevant recruitment website that was responsive to needs of young adults. Investing in real time tracking of recruitment yields was helpful to guide recruitment decision making over time. These steps, coupled with adequate financial resources budgeted for recruitment in the grant application, are recommended for successful recruitment of historically challenging populations in future trials.

## Conclusions

Recruitment of 599 normal weight and overweight 18- to 35-year-old adults for a weight gain prevention randomized controlled trial cost about $233 per participant enrolled (not including personnel time) and required 19 months of sustained efforts. The most successful methods of recruitment in SNAP were mass mailing followed by email. The most cost-effective method with high yield was email. Investing in formative research and professional marketing services prior to launching recruitment efforts was useful in guiding our efforts. Successful channels were prioritized through careful, real-time tracking of recruitment yields. Thus, challenging populations can be recruited for clinical trials provided that adequate time, attention and resources are devoted to this task.

## Abbreviations

BMI: body mass index
IRB: institutional review board
NC: North Carolina
OR: odds ratio
RI: Rhode Island
SNAP: Study of Novel Approaches to Prevention
UNC: University of North Carolina.
